# Telemedicine to Decrease Personal Protective Equipment Use and Protect Healthcare Workers

**DOI:** 10.5811/westjem.2020.8.47802

**Published:** 2020-09-24

**Authors:** Ryan Ribeira, Sam Shen, Patrice Callagy, Jennifer Newberry, Matthew Strehlow, James Quinn

**Affiliations:** Stanford University School of Medicine, Department of Emergency Medicine, Palo Alto, California

## TO THE EDITOR

Infectious disease outbreaks, such as coronavirus disease 2019 (COVID-19), place tremendous strain on availability of personal protective equipment (PPE) and frontline healthcare providers. Readily available PPE can substantially reduce the rate of infection in healthcare workers and the spread of the illness.^1^,^2^ The lack of adequate PPE places providers at increased risk of infection, increases healthcare worker stress, and decreases staffing as providers fall ill. We know that inadequate PPE and risk of becoming infected are primary concerns of healthcare providers during pandemics, serving as key drivers in their willingness to work.^3^,^4^ Therefore, it is imperative that efforts are undertaken to minimize the threat facing them and their families.^5^ Here, we describe an emergency department (ED) effort to safely limit PPE use and decrease the risk of illness to providers by implementing telemedicine to care for patients already within our department walls.

## LEVERAGING IN-ROOM TELEMEDICINE FOR INFLUENZA-LIKE ILLNESS PATIENTS

Patients approaching our ED are screened outside by a nurse in full PPE for influenza-like illness symptoms. For those who screen positive, a tele-registration protocol is initiated. Using a secure device, a patient’s photo identification and phone number are forwarded to registration staff, who then complete the registration process remotely by phone. Those with mild symptoms are directed to a drive-through, where a telemedicine cart facilitates an encounter with a physician who determines the need for a swab. A nurse in PPE moves from vehicle to vehicle performing swabs and providing standardized discharge instructions.

Patients with severe symptoms are redirected to an alternate ED entrance, which leads into an anteroom that immediately separates potentially positive patients from the general ED population. ED rooms are outfitted with a wall-mounted television and wide-angle camera with directional speaker system. After trialing this system, we found that it was more efficient and effective to use iPads (Apple Inc, Cupertino, CA) on rolling stands because they worked more reliably, were easier for physicians to use, and required fewer room entries for configuration. Following a successful pilot, each ED room and clinician work area was outfitted with an iPad and stand for a total of 100 units across both our adult and pediatric EDs.

This system has the additional benefit of being relatively cost efficient, with each iPad and stand costing $1099.40 per unit. This means for an average ED with approximately 30 beds and four physician/nurse work areas it would cost $37,379.60 for a similar telemedicine system. Optimal utilization of this system requires synchronized team communication. For most encounters, the number of providers required to enter the patient room can be reduced to one. The rest of the care team (including trainees, nurses, consultants, and interpreters) can observe and engage via telemedicine. In addition, critical care physicians can provide input remotely during high exposure-risk resuscitations.

## SUMMARY

Telemedicine saves at least one to two interactions per patient that would otherwise require PPE. While this strategy minimizes unnecessary exposures for our healthcare workers, they are not restricted from physically assessing patients when deemed necessary. The risks and benefits of physical interaction requiring PPE are left to provider discretion, although we found that most COVID-19 patients under investigation at our ED can be managed through telemedicine.

Research has shown that telemedicine is safe and effective, and that the degree of illness severity can be assessed without direct interaction.^6^ While direct auscultation of the chest cannot be performed remotely, the value of this exam for these patients is debatable. Auscultation alone has poor interobserver agreement and can miss 50% of pneumonias, which are better predicted by oxygen saturation less than 95%, fever, and tachycardia, with the gold standard being chest radiograph (CXR).^7^–^10^ Respiratory status can be assessed reliably by talking with the patient, evaluating his or her history, and observing for objective signs of respiratory compromise, with the addition of a CXR when indicated.

Our ED had a sophisticated telemedicine system built into every ED room prior to COVID, yet we found that a low-cost iPad-based system was more effective and could potentially be quickly deployed in other settings to conserve valuable PPE and prioritize healthcare worker safety. During the COVID-19 pandemic, healthcare systems and providers must rapidly innovate and disseminate practices that strengthen our crisis management capabilities.
